# Thermosensitive Alginate/Carrageenan–Stearic Acid Dissolving Microneedles Incorporating Liposome-Coated Hollow Mesoporous Silica Nanoparticles for Controlled Caffeine Delivery

**DOI:** 10.3390/ijms27157067

**Published:** 2026-08-06

**Authors:** Nattanida Thepphankulngarm, Namon Hirun, Pakorn Kraisit

**Affiliations:** Thammasat University Research Unit in Smart Materials and Innovative Technology for Pharmaceutical Applications (SMIT-Pharm), Faculty of Pharmacy, Thammasat University, Pathumthani 12120, Thailand; nattanida.t@gmail.com (N.T.); namon.hi@tu.ac.th (N.H.)

**Keywords:** caffeine, dissolving microneedles, hollow mesoporous silica nanoparticles, liposomes, alginate, carrageenan

## Abstract

Caffeine is a promising active for topical pharmaceutical and cosmetic applications; however, its hydrophilic nature can limit passive transport across the stratum corneum and reduce local retention. Although nanoparticle-loaded dissolving microneedles have been explored, few systems combine a hollow porous carrier, a lipid coating, and a polysaccharide matrix within a single controlled-release platform. This study developed thermosensitive dissolving microneedles (DMNs) incorporating caffeine-loaded liposome-coated hollow mesoporous silica nanoparticles (ULp-Caf@HMSNs). Sodium alginate–κ-carrageenan matrices, with and without stearic acid modification, were evaluated for their effects on thermal behavior, mechanical strength, and caffeine release. HMSNs showed a hollow mesoporous structure and high specific surface area, while FTIR supported successful incorporation of the formulation components. Liposome coating increased particle size while maintaining nanoscale dimensions. Hot-stage microscopy showed temperature-dependent structural changes at approximately 33–35 °C, with lower apparent transition temperatures in stearic acid-containing formulations. All DMNs exhibited compression forces comparable to mechanically competent DMNs, with NaAlg–κ-car systems reaching 14.3–15.1 N per array. Free-caffeine DMNs followed non-Fickian release, whereas ULp-Caf@HMSN-containing formulations followed Higuchi diffusion-controlled release and showed slower caffeine release. Stearic acid did not consistently improve release performance. The novelty of this work lies in integrating a hollow silica reservoir, a liposomal coating, and an alginate–κ-carrageenan microneedle matrix within one platform. Overall, the system provides a basis for further evaluation in controlled topical or transdermal caffeine delivery.

## 1. Introduction

Caffeine (Caf) is a naturally occurring methylxanthine with several biological activities that support its use in pharmaceutical and topical formulations. At physiologically relevant concentrations, its actions are primarily associated with the antagonism of adenosine receptors, whereas inhibition of phosphodiesterase generally becomes more prominent at higher concentrations. Phosphodiesterase inhibition reduces the degradation of cyclic adenosine monophosphate (cAMP), thereby increasing intracellular cAMP and promoting downstream signaling. In adipose tissue, this mechanism can stimulate lipolysis and the breakdown of stored triglycerides, which provides a rationale for the inclusion of Caf in topical formulations intended to improve the appearance of cellulite [1]. Caf also exhibits antioxidant activity and has been reported to protect cells against oxidative and ultraviolet-induced damage, although these effects depend on the concentration and experimental conditions [1]. Its potential application in hair-loss management has also received considerable attention. In an ex vivo human hair-follicle model, Fischer et al. demonstrated that Caf at concentrations of 0.001% and 0.005% counteracted testosterone-induced growth suppression and significantly stimulated hair-follicle growth, with increased keratinocyte proliferation confirmed by Ki-67 staining [2]. Subsequent reviews have identified Caf as one of the most extensively investigated plant-derived compounds for topical management of androgenetic alopecia, while emphasizing that its clinical effectiveness remains dependent on dose, formulation, and delivery to the hair follicle [3,4]. Although Caf can penetrate human skin and preferentially enter through the follicular pathway, its hydrophilic nature limits passive partitioning through the lipid-rich stratum corneum. This limitation supports the development of delivery systems that enhance follicular deposition and provide controlled and sustained Caf release.

The clinical value of topical Caf depends not only on its biological activity but also on how much reaches the hair follicle and how long it remains available at the application site. Most conventional Caf products are formulated as shampoos, lotions, or hydroalcoholic solutions. Studies have shown that Caf from shampoo formulations can enter the hair follicles within minutes, with the follicular route contributing substantially during the early stage of absorption [5,6]. However, rinse-off products have only a brief contact time, while lotions and solutions may be removed from the scalp or diffuse away relatively quickly. A controlled-release system may therefore help maintain local Caf availability for a longer period and reduce the rapid loss of freely dissolved drug. This could be particularly relevant for follicular delivery, where rapid initial uptake does not necessarily result in prolonged retention. Nevertheless, the clinical advantage of sustained Caf release has not yet been established and should be confirmed in future skin-deposition and efficacy studies.

Liposomes (ULps) are biocompatible carriers with high encapsulation efficiency. However, their relatively rigid bilayer structure may limit penetration into deeper skin layers [7]. Liposomes address this limitation by increasing bilayer flexibility and improving skin penetration, particularly through the transappendageal pathway [8,9]. Mesoporous silica nanoparticles (MSNs) have attracted considerable attention as drug-delivery carriers because of their large surface area, high pore volume, stability, and biocompatibility [10]. These properties can improve drug solubility, prolong circulation time, enhance drug accumulation at target sites, and reduce undesirable effects [11]. Nevertheless, conventional MSNs may have limited drug-loading capacity because of their solid internal structure and restricted pore volume. Hollow mesoporous silica nanoparticles (HMSNs) were therefore developed to provide a larger internal space, higher drug-loading capacity, and more efficient diffusion of bioactive compounds [10,12,13]. To enhance transdermal drug delivery, nanoparticle-based systems have also been combined with physical enhancement methods, including microneedles (MNs), iontophoresis, electroporation, and ultrasound [14,15,16,17]. More recently, MSNs with tunable pore structures have been incorporated into dissolving microneedle patches to provide an intradermal nanoparticle reservoir and sustained cargo release. This approach confirms the value of combining porous nanocarriers with MNs, although the effects of an additional lipid coating and a thermoresponsive polysaccharide matrix remain insufficiently explored [18]. Among these approaches, MNs can markedly increase skin permeability [19]. Typically ranging from 25 to 1000 μm in length, MNs penetrate the stratum corneum (SC) while generally avoiding the deeper, pain-sensitive layers of the skin. Several types of MNs have been developed, including solid MNs that create microchannels [20,21], coated MNs that deliver drugs from their surface coatings [22,23,24], hollow MNs that allow the injection of liquid formulations [23,24,25], and dissolving MNs that dissolve after insertion and reduce concerns related to sharp-waste disposal. Dissolving microneedles (DMNs) are particularly attractive for enhancing the delivery of hydrophilic molecules across the SC. Recent studies have further highlighted the potential of DMNs to bypass the stratum corneum and deliver therapeutic agents directly into the viable skin layers. However, their performance remains strongly influenced by drug-loading capacity, dose uniformity, mechanical strength, dissolution behavior, and the matrix’s ability to provide controlled release [26,27]. By forming transient microchannels and subsequently dissolving within the hydrated skin environment, DMNs can enhance local drug deposition while avoiding the generation of sharp waste. However, their practical performance depends strongly on the mechanical integrity, thermal responsiveness, and release characteristics of the polymeric matrix. MNs have been fabricated from various polymers, including polyvinyl alcohol (PVA). However, PVA-based MNs may have insufficient mechanical strength to penetrate the skin effectively without further formulation optimization or reinforcement.

Polysaccharide-based gels have attracted considerable interest as drug-delivery matrices because their hydrated polymer networks can protect incorporated drugs and regulate drug diffusion and release. Natural polysaccharides, including carrageenan, alginate, chitosan, agarose, pectin, and cellulose derivatives, have been developed as hydrogels, beads, films, and in situ gelling systems for oral, topical, mucosal, and other local delivery applications. Carrageenan is particularly useful because its sulfated polysaccharide chains undergo ion- and temperature-dependent gelation, allowing it to be fabricated into hydrogels, microparticles, microspheres, aerogels, and three-dimensional printed matrices for controlled drug delivery [28]. Carrageenan-based hydrogels have also been explored for drug delivery, tissue engineering, and wound healing, although their mechanical properties often require improvement through crosslinking or blending with other polymers [29]. The versatility of polysaccharide gels is further illustrated by a pH-responsive hydrogel composed of hydroxypropyl-β-cyclodextrin, agarose, and poly(methacrylic acid), which was designed to protect capecitabine under gastric conditions and promote its controlled oral release at intestinal pH [30]. Polysaccharide-based gel matrices can also be combined with nanoparticles to protect incorporated therapeutic agents, provide antimicrobial activity, and enable controlled or localized drug release, particularly in wound-healing and transdermal delivery applications [31].

Sodium alginate (NaAlg) is hydrophilic, highly absorbent, and biocompatible. It forms hydrogels through cross-linking and gelation, making it suitable for drug-delivery systems designed to retain moisture and provide controlled drug release. NaAlg can improve formulation stability, reduce skin irritation, and remain compatible with the mildly acidic to neutral pH range commonly used in topical formulations [32,33,34]. Its established use in FDA-regulated pharmaceutical and biomedical products further supports its potential as a biomaterial, while some NaAlg-based formulations have remained stable for up to 90 days [35]. Modified NaAlg may also exhibit thermosensitive behavior, with a sol-to-gel transition occurring at approximately 35 °C [36]. However, its relatively low mechanical strength and limited ability to encapsulate certain drugs, particularly hydrophobic compounds, may restrict its use in applications requiring high moisture resistance [37,38].

κ-Carrageenan (κ-car) has been investigated as a complementary polymer to improve the properties of NaAlg-based matrices. It offers biocompatibility, controlled-release properties, and stability under the mildly acidic conditions of the stratum corneum (SC; pH 4.5–5.3) [37,38]. Prasetyaningrum et al. reported that increasing the proportion of κ-car in NaAlg films improved tensile strength and Young’s modulus, possibly through stronger ionic interactions within the polymer network [39]. κ-Car also exhibits thermoreversible gelation, which may support the development of thermosensitive MNs [39]. Accordingly, NaAlg–κ-car composites are promising candidates for mechanically robust and thermoresponsive DMN matrices.

Despite these advantages, NaAlg–κ-car composites may have limited flexibility and ductility, which can affect their mechanical performance and interaction with the skin. Incorporating fatty acids, such as stearic acid (SA), into the composite matrix may improve structural properties, moisture resistance, and permeability [40,41]. Combining hydrophobic lipids with hydrophilic biopolymers can strengthen barrier properties and reduce moisture uptake. Because of its long C18 hydrocarbon chain, SA may increase hydrophobic interactions within the matrix and modify polymer packing and intermolecular interactions [42]. Thus, incorporating SA into NaAlg–κ-car composites to form alginate stearate may alter the hydrophobicity, mechanical properties, thermal behavior, and drug-release characteristics of the matrix. The incorporation of ULp-Caf@HMSNs within DMNs, together with SA modification, was therefore investigated as a strategy to modulate Caf release and improve the physicochemical and mechanical properties of the formulation.

Nanoparticle-loaded DMNs have been investigated as a way to combine the skin-penetrating ability of MNs with the drug-loading and release properties of nanocarriers. PLGA nanoparticles, for example, have been incorporated into alginate and other polymeric MNs to improve drug deposition, mechanical strength, and sustained release. Nanocrystals have also been used to increase the apparent solubility and loading of poorly water-soluble drugs [43,44]. More recently, MSNs with different pore sizes have been localized within dissolving microneedle tips to enable intradermal nanoparticle deposition and tunable drug release [18]. Despite these advances, most reported systems rely on a single type of nanocarrier and are not designed to address several formulation and delivery challenges at the same time, such as limited loading capacity, premature drug release, transport across both hydrated and lipid-rich skin regions, and the need to maintain suitable mechanical and thermal properties of the microneedle matrix.

In our previous study, Caf-loaded HMSNs were coated with ULps to form ULp-Caf@HMSNs. The hollow mesoporous structure served as a reservoir for Caf, while the lipid coating slowed drug release and improved follicular penetration [45]. However, the particulate formulation itself did not provide a solid, self-applicable platform capable of creating microchannels across the stratum corneum and delivering the nanocarriers into the skin. The present study therefore incorporates ULp-Caf@HMSNs into DMNs prepared from NaAlg and κ-car. In this system, the HMSNs provide a porous Caf reservoir, the liposomal coating introduces an additional diffusion barrier and may improve interactions with lipid-rich skin structures, and the MNs are designed to create transient microchannels across the stratum corneum. NaAlg and κ-car were selected to form a biocompatible and hydrated matrix with temperature-responsive behavior and sufficient mechanical strength for insertion. SA modification was also examined to determine whether increased hydrophobic interactions could alter the thermal, mechanical, and release characteristics of the polysaccharide matrix. Accordingly, the present work goes beyond the simple incorporation of nanoparticles into DMNs by combining a hollow silica reservoir, a lipid coating, a thermoresponsive polysaccharide matrix, and fatty-acid modification within a single delivery platform. To the best of our knowledge, this specific combination has not previously been evaluated for controlled Caf delivery.

Accordingly, this study aimed to develop temperature-responsive DMNs containing ULp-Caf@HMSNs for controlled Caf delivery. The nanocarriers were incorporated into NaAlg–κ-car matrices, with and without SA modification, to examine how the matrix composition affected microneedle morphology, thermal behavior, mechanical strength, and drug release. MNs containing free Caf were included as control formulations to distinguish the contribution of the nanocarrier system from that of the polysaccharide matrix. The HMSNs were characterized in terms of morphology, porosity, particle size, and surface charge, while the resulting MNs were evaluated by FTIR spectroscopy, microscopy, hot-stage analysis, compression testing, and in vitro release studies. This approach allowed the effects of the HMSNs, liposomal coating, and SA modification on the overall performance of the DMNs to be compared.

## 2. Results and Discussion

### 2.1. Characterization of HMSNs and Physical Properties of NaAlg-κ-Car/Caf, SA-Alg-κ-Car/Caf, NaAlg-κ-Car/ULp-Caf@HMSNs, and SA-Alg-κ-Car/ULp-Caf@HMSNs in the Complex Gels and DMNs

**Figure 1:**
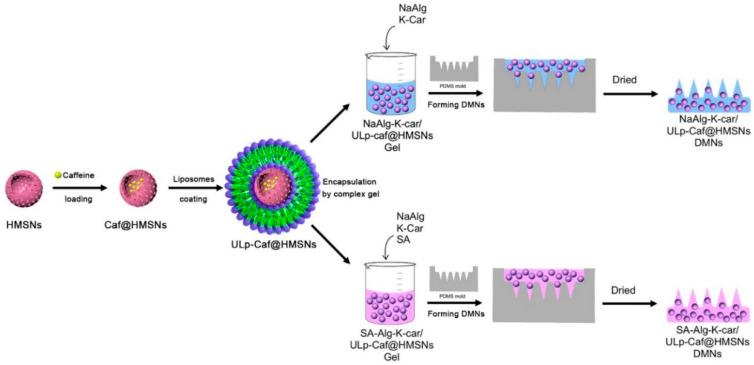
Schematic diagram illustrating the synthesis of NaAlg-κ-car/ULp-Caf@HMSNs DMNs and SA-Alg-κ-car/ULp-Caf@HMSNs DMNs.

Figure 1 illustrates the preparation protocol for NaAlg-κ-car/ULp-Caf@HMSNs and SA-Alg-κ-car/ULp-Caf@HMSNs DMNs. The process commenced with the surface modification of Caf@HMSNs by coating them with ULps to produce ULp-Caf@HMSNs. These modified particles were then incorporated into NaAlg-κ-car and SA-Alg-κ-car hybrid matrices to prepare NaAlg-κ-car/ULp-Caf@HMSNs and SA-Alg-κ-car/ULp-Caf@HMSNs hydrogels, respectively. The hydrogels were subsequently cast into PDMS molds to fabricate NaAlg-κ-car/ULp-Caf@HMSNs DMNs and SA-Alg-κ-car/ULp-Caf@HMSNs DMNs.

**Figure 2:**
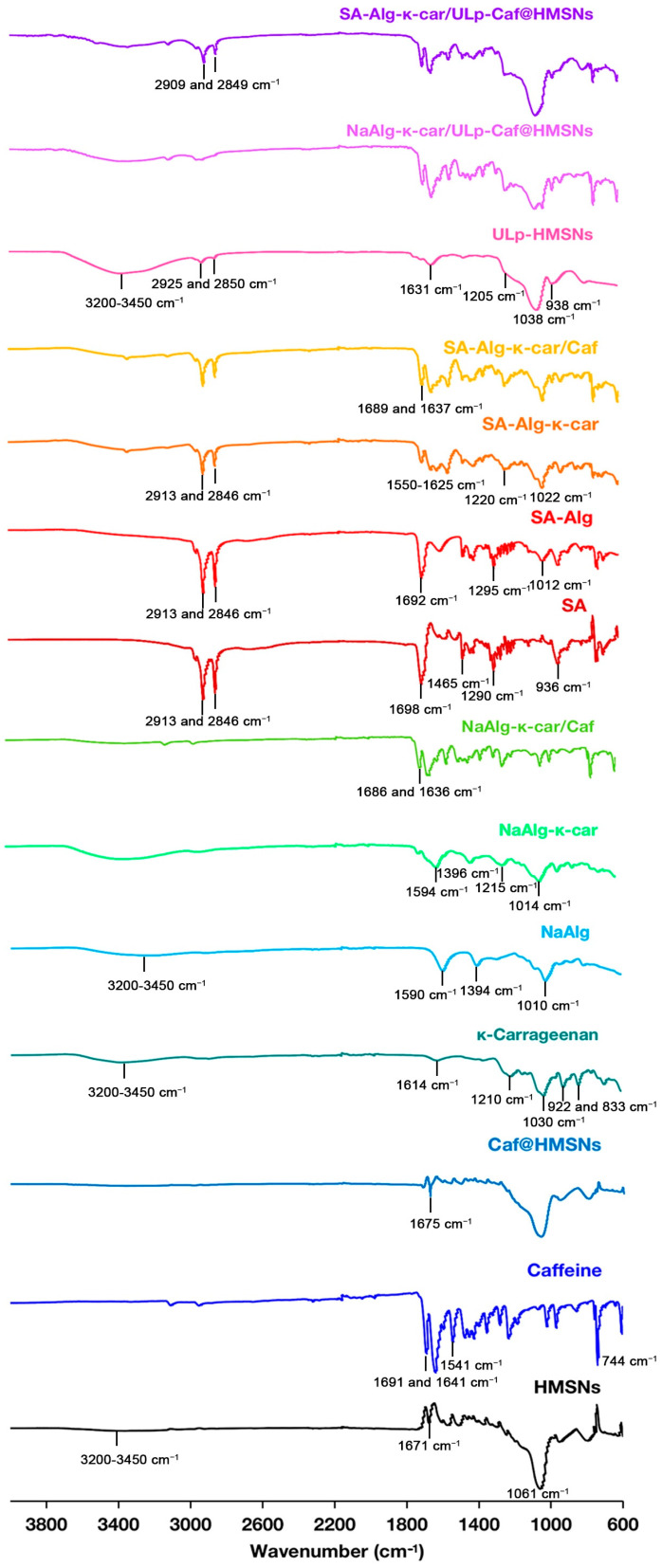
FTIR spectra of the samples.

FTIR spectroscopy was used to examine the characteristic functional groups of the raw materials, nanoparticles, and composite formulations, as shown in Figure 2 and summarized in Table 1. Pristine HMSNs showed a broad O–H stretching band at 3200–3450 cm^−1^ and an H–O–H bending band near 1671 cm^−1^, which were attributed to surface silanol groups and adsorbed water. The strong band at 1061 cm^−1^ was assigned to the asymmetric stretching vibration of Si–O–Si, confirming the formation of the silica framework [46,47]. After Caf loading, this band shifted to approximately 1038 cm^−1^. This shift indicates that the local environment of the silica network was altered, most likely because of hydrogen-bonding interactions between the surface silanol groups of HMSNs and the carbonyl or nitrogen-containing groups of Caf. Confinement of Caf within the mesopores and partial overlap with nearby Caf-related vibrations may also have contributed to the observed shift. However, the Si–O–Si band remained clearly detectable, indicating that the silica framework was preserved after drug loading. In addition, the Caf-associated carbonyl band near 1675 cm^−1^ was retained with reduced intensity, supporting the incorporation of Caf into the HMSNs without detectable degradation of its chemical structure. The asymmetric and symmetric C-H stretching vibrations of methylene groups were identified as new bands in the ULp-HMSN spectrum at 2920 and 2860 cm^−1^. The carbonyl vibrations were identified by the bands at 1640 and 1370 cm-1. The band at 1095 cm^−1^ was attributed to –CH_2_–O–CH_2_– stretching [48]. Oleic acid exhibited distinctive bands at 1743 cm^−1^, attributed to C=O stretching, and at 1464 and 944 cm^−1^, associated with in-plane and out-of-plane O–H bending vibrations, respectively [49]. Phospholipon^®^ 90 G was successfully incorporated into the formulation, as evidenced by the diagnostic bands at 1236 cm^−1^ (P=O stretching), 1088 cm^−1^ (P–O–C stretching), and 970 cm^−1^ (–N(CH_3_)_3_^+^ stretching). For pure Caf, the characteristic bands at 1691 and 1641 cm^−1^ were assigned to carbonyl stretching coupled with C=N stretching and vibrations of the xanthine ring, respectively. An additional band at 744 cm^−1^ was attributed to out-of-plane bending vibration of the xanthine ring [50]. κ-Car showed intense O–H stretching bands at 3200–3450 cm^−1^, together with an H–O–H bending band attributed to absorbed water at 1614 cm^−1^ and an S=O stretching band associated with sulfate ester groups (SO_3_^−^) at 1210 cm^−1^. The bands at 1030 and 922 cm^−1^ were attributed to C–O–C stretching and bending vibrations of the glycosidic linkages and the 3,6-anhydro bridge, respectively. The band at 833 cm^−1^ was ascribed to C–O–S stretching in 3,6-anhydro-D-galactose [51,52]. In NaAlg, a prominent O–H stretching band was identified around 3200–3450 cm^−1^, whereas asymmetric and symmetric COO- stretching vibrations were noted at 1590 and 1394 cm^−1^, respectively. The prominent band at 1010 cm^−1^ was attributed to C–O–C and C–O stretching vibrations of the polysaccharide backbone [53]. In the NaAlg-κ-car hybrid spectrum, characteristic signals of both biopolymers were maintained, with minor shifts suggesting intermolecular interactions between the sulfate groups of κ-car and the alginate matrix. In addition, the broad O–H stretching band at 3250–3400 cm^−1^ suggested hydrogen bonding between the polymer chains [54]. Following Caf incorporation into NaAlg-κ-car to form NaAlg-κ-car/Caf, the bands at 1636 and 1686 cm^−1^ remained detectable, suggesting that Caf retained its chemical structure without undergoing significant chemical interactions with the polymer network. SA displayed prominent C–H stretching bands at 2846 and 2913 cm^−1^, corresponding to symmetric and asymmetric –CH_2_– vibrations, respectively. The C=O stretching band appeared at 1698 cm^−1^. Additional bands at 1465, 1290, and 936 cm^−1^ were attributed to C–H bending, C–O stretching, and C–O stretching coupled with C–C–H and C–O–H deformation vibrations, respectively [55]. When SA was combined with NaAlg to form the SA-Alg composite, FTIR analysis revealed slight shifts in band positions, indicating physical interactions between the SA chains and the alginate matrix. After κ-car and Caf were added to produce SA-Alg-κ-car/Caf, similar spectral features were retained, further supporting the presence of weak intermolecular interactions. Finally, the incorporation of ULp-Caf@HMSNs into the NaAlg-κ-car and SA-Alg-κ-car hydrogels produced NaAlg-κ-car/ULp-Caf@HMSNs and SA-Alg-κ-car/ULp-Caf@HMSNs, respectively. These composite systems retained the characteristic spectral features of their individual components, supporting the successful formation of the multicomponent drug-delivery systems.

**Figure 3:**
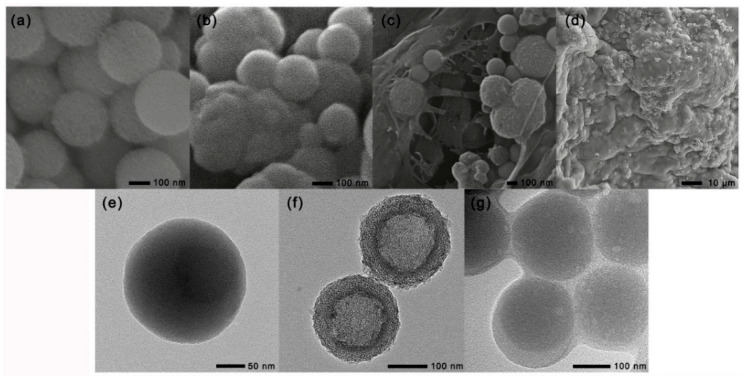
FE-SEM images of (**a**) HMSNs, (**b**) ULp-Caf@HMSNs, (**c**) NaAlg-κ-car/ULp-Caf@HMSNs, and (**d**) SA-Alg-κ-car/ULp-Caf@HMSNs. TEM images of (**e**) SiO2@CTAB-SiO2 Core/Shell Nanoparticles, (**f**) HMSNs after etching, and (**g**) ULp-Caf@HMSNs.

SEM images of HMSNs, ULp-Caf@HMSNs, NaAlg-κ-car/ULp-Caf@HMSNs, and SA-Alg-κ-car/ULp-Caf@HMSNs are shown in Figure 3a–d. The synthesized nanomaterials exhibited a spherical morphology and a relatively uniform particle-size distribution. Pristine HMSNs (Figure 3a) had diameters of 190–230 nm, confirming their nanoscale dimensions [56]. Following liposome coating, the particle diameter increased by approximately 50–70 nm, resulting in particle sizes of 220–260 nm (Figure 3b) [45]. The composite materials (Figure 3c,d) exhibited a distinctive ravine-like surface topography, which may increase the available surface area. Their fibrous network morphology may reflect intermolecular interactions between the NaAlg and κ-car chains, including hydrogen bonding and van der Waals forces [57,58]. The incorporation of SA resulted in subtle changes in surface roughness. Discrete surface features were also observed throughout the gel matrix; however, further compositional analysis would be required to confirm whether these features corresponded to SA. These morphological changes may be associated with changes in the mechanical and thermal properties of the matrix. TEM analysis (Figure 3e–g) provided further structural information. Before etching, dense silica cores appeared as electron-dense regions (Figure 3e). After etching, hollow cavities surrounded by mesoporous silica shells were clearly observed (Figure 3f), indicating successful template removal. Following liposome coating, an additional electron-lucent layer was observed around the HMSNs (Figure 3g), supporting the successful deposition of a lipid layer on the particle surface [59].

The physicochemical characterization data presented in Table 2 show the zeta potential, PDI, and mean particle diameter of the HMSN and ULp-Caf@HMSN systems. The uncoated HMSNs had an average particle diameter of approximately 198.2 nm, which increased to 243.9 nm after liposome coating in the ULp-Caf@HMSN formulation [60]. This corresponds to an increase of approximately 45.7 nm, which was attributed to the lipid-based coating. This size enhancement confirms successful liposome coating while maintaining particle dimensions within the nanoscale range commonly reported for drug-delivery systems. The liposomal coating may improve colloidal properties and provide an additional lipid-based barrier that can modulate Caf release. The HMSNs and ULp-Caf@HMSNs showed PDI values of 0.487 and 0.422, respectively, indicating moderate size heterogeneity. Their zeta potentials ranged from approximately −18 to −25 mV, confirming that both formulations carried negatively charged surfaces. The surface silanol groups of HMSNs become deprotonated at neutral pH, resulting in a negative surface charge. Because mesoporous silica has a point of zero charge in the acidic range (approximately pH 2–3), it carries a net negative charge under physiological conditions. ULp-Caf@HMSNs showed a more negative surface charge, which was likely associated mainly with the ionization of oleic acid incorporated into the lipid layer. Oleic acid contains carboxylic acid groups that deprotonate at physiological pH and contribute additional negative charges. Although phosphatidylcholine is zwitterionic and contains both positively charged choline and negatively charged phosphate groups, it generally has an overall near-neutral charge and may primarily influence the organization of the lipid coating rather than directly account for the increased negative zeta potential [45].

The encapsulation efficiency (%EE) and loading capacity (%LC) of Caf@HMSNs in an aqueous medium were 32.11% and 24.31%, respectively. Caf is primarily incorporated into HMSNs through physisorption, involving hydrogen bonding between its carbonyl and imide nitrogen groups and the surface silanol groups of the mesoporous silica framework. Van der Waals forces and electrostatic interactions may provide additional stabilization, consistent with the adsorption mechanisms reported for other mesoporous silica systems. To maintain a consistent Caf concentration across all DMN formulations, including NaAlg-κ-car/Caf, SA-Alg-κ-car/Caf, NaAlg-κ-car/ULp-Caf@HMSNs, and SA-Alg-κ-car/ULp-Caf@HMSNs, the same previously characterized batch of Caf@HMSNs was used. The Caf-loading characteristics, including an incorporated amount of 32.11 mg, were reported in our previous study [10] and were therefore not reassessed in the present study. Conventional MSNs typically show drug-loading capacities of 200–300 mg g^−1^, although values approaching 600 mg g^−1^ have been reported [61]. When expressed as percentages, loading capacities generally range from 20% to 30%, with exceptional values reaching approximately 37.5%. Thus, the previously established loading capacity of Caf@HMSNs (24.31%) falls within the commonly reported range for mesoporous silica-based nanocarriers.

**Figure 4:**
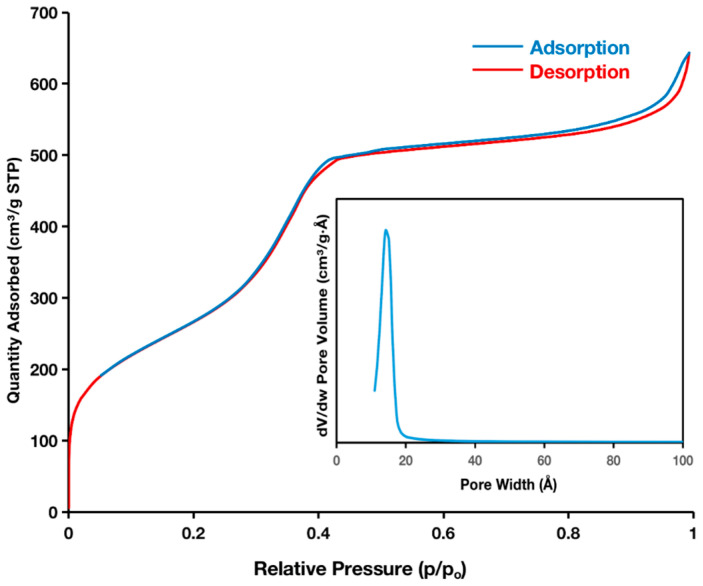
Nitrogen adsorption–desorption isotherms of HMSNs. Insets show the corresponding pore size distributions. The desorption branch is represented by the upper red curve, while the adsorption branch is represented by the lower blue curve.

Nitrogen physisorption measurements were conducted to assess the textural characteristics of HMSNs, including pore width, pore volume, and specific surface area. As shown in Figure 4, the adsorption–desorption isotherm exhibited a Type IV profile according to the IUPAC classification, which is characteristic of mesoporous materials [62]. The inset of Figure 4 shows the pore-size distribution, with an average pore diameter of 1.8 nm, indicating that the pores were close to the boundary between the microporous and mesoporous ranges within the silica shell. The prepared HMSNs exhibited a highly porous structure suitable for Caf encapsulation, with a pore volume of 1.02 mL g^−1^ and a BET surface area of 1015.44 m^2^ g^−1^. Compared with conventional MSNs, which often exhibit pore volumes ranging from 0.47 to 0.69 mL g^−1^ [63], the HMSNs developed in this study showed a substantially higher pore volume. This value was close to those reported for other hollow mesoporous silica systems, which range from 1.14 to 1.82 mL g^−1^ [63]. The greater pore volume of HMSNs than that of solid MSNs may be attributed to the hollow internal cavity in addition to the mesoporous shell network, providing greater internal space for drug loading. The high surface area, large pore volume, and hollow structure of the HMSNs provide ample internal space and adsorption sites for Caf loading. The same previously characterized Caf@HMSN batch was used in this study. This batch had an encapsulation efficiency of 32.11% and a loading capacity of 24.31%, corresponding to an incorporated Caf amount of 32.11 mg [10]. Caf is likely retained mainly through physical adsorption, including hydrogen bonding with surface silanol groups, as well as van der Waals forces and other weak intermolecular interactions. The relatively narrow average pore diameter of approximately 1.8 nm may restrict Caf diffusion through the silica shell and thereby contribute to the slower release from formulations containing HMSNs. The loading capacity falls within the range commonly reported for mesoporous silica-based nanocarriers [61]. Because these loading data were obtained in our previous study and were not reassessed in the present work, the proposed relationship between pore structure and release behavior should be regarded as a plausible interpretation rather than a definitive conclusion.

**Figure 5:**
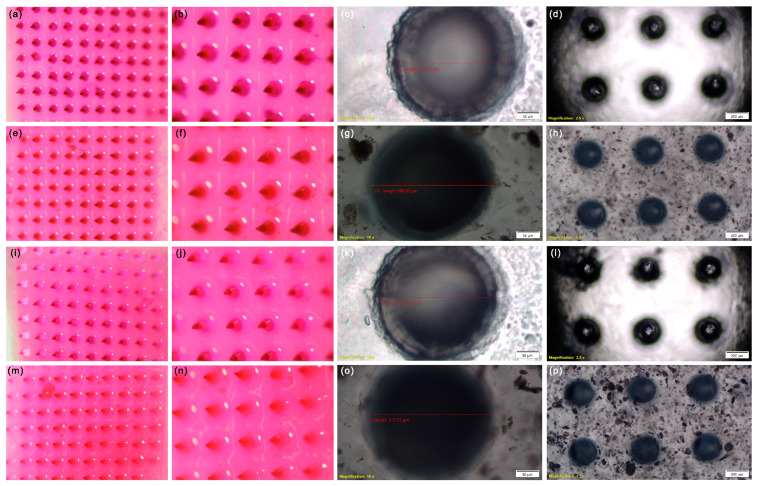
Stereo microscope images illustrate the physical morphology and array arrangement of (**a**,**b**) NaAlg-κ-car/Caf DMNs, (**e**,**f**) SA-Alg-κ-car/Caf DMNs, (**i**,**j**) NaAlg-κ-car/ULp-Caf@HMSNs DMNs, and (**m**,**n**) SA-Alg-κ-car/ULp-Caf@HMSNs DMNs. Microscope images show the needle diameter and interbase spacing for (**c**,**d**) NaAlg-κ-car/Caf DMNs, (**g**,**h**) SA-Alg-κ-car/Caf DMNs, (**k**,**l**) NaAlg-κ-car/ULp-Caf@HMSNs DMNs, and (**o**,**p**) SA-Alg-κ-car/ULp-Caf@HMSNs DMNs, respectively, with magnifications of 10× for (**c**,**g**,**k**,**o**) and 2.5× for (**d**,**h**,**l**,**p**).

The morphological evaluation showed that all four formulations—NaAlg-κ-car/Caf DMNs, SA-Alg-κ-car/Caf DMNs, NaAlg-κ-car/ULp-Caf@HMSNs DMNs, and SA-Alg-κ-car/ULp-Caf@HMSNs DMNs—formed well-defined microneedle arrays with uniform and orderly arrangements, as shown in Figure 5a–p. The statistical analysis presented in Table 3 revealed significant differences in microneedle morphology and dimensions among the formulations. The measured needle diameters and base-to-base spacings closely matched the specifications of the PDMS mold, confirming successful replication of the master-template geometry.

**Figure 6:**
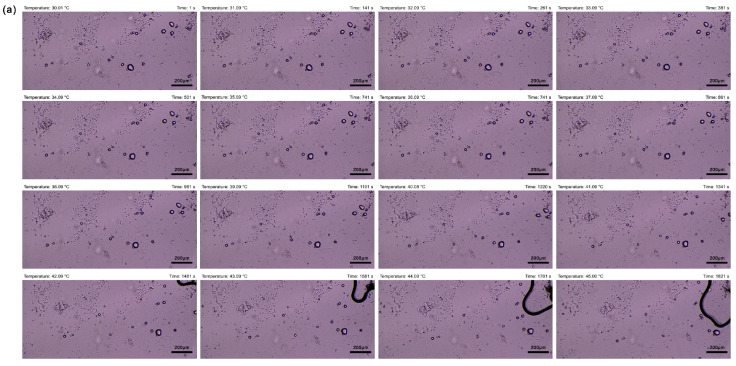

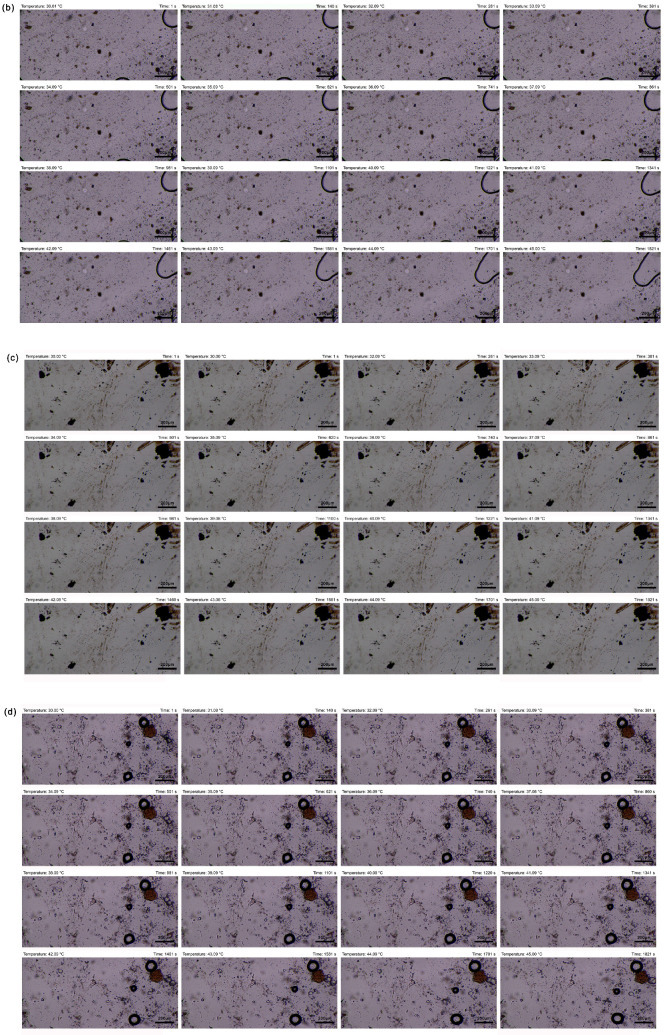

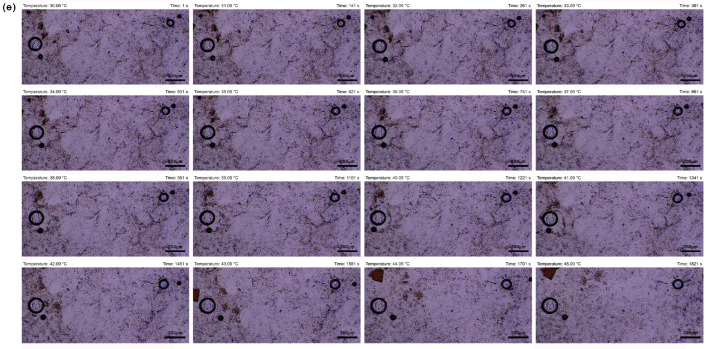
HSM images of (**a**) NaAlg-κ-car/Caf, (**b**) SA-Alg-κ-car/Caf, (**c**) ULp-Caf@HMSNs, (**d**) NaAlg-κ-car/ULp-Caf@HMSNs, and (**e**) SA-Alg-κ-car/ULp-Caf@HMSNs were recorded over a temperature range of 31–45 °C.

Thermal characterization using HSM revealed distinct thermal-transition behaviors within the physiologically relevant temperature range of 31–45 °C, as shown in Figure 6a–e. NaAlg-κ-car/Caf showed an apparent transition temperature of 35.09 ± 0.5 °C, which was comparable to that of the NaAlg-κ-car/ULp-Caf@HMSNs DMN formulation. This similarity suggests that nanoparticle incorporation did not markedly alter the thermal behavior of the alginate–carrageenan matrix [64]. In contrast, the SA-containing formulations showed lower apparent transition temperatures. SA-Alg-κ-car/Caf and SA-Alg-κ-car/ULp-Caf@HMSNs exhibited values of 34.09 ± 0.5 °C and 33.09 ± 0.5 °C, respectively. The lower apparent transition temperatures observed in the SA-containing formulations indicate that SA altered the thermal behavior of the polysaccharide matrix. One possible explanation is that the long hydrophobic chains of SA partially interfere with hydrogen bonding and polymer-chain packing between alginate and κ-car, resulting in a less compact matrix. Similar effects of fatty-acid incorporation on polymer organization and thermal behavior have been reported in polysaccharide- and protein-based films [40,41,42]. However, this explanation remains a hypothesis because polymer crystallinity and molecular organization were not directly examined in the present study. The alginate–carrageenan composite showed a higher apparent thermal-transition temperature, which may be related to closer polymer-chain packing and stronger hydrogen-bonding interactions than those in the SA-modified matrices. Notably, the incorporation of HMSNs did not significantly alter the thermal-transition behavior of either formulation. This finding suggests good thermal compatibility between the silica nanocarriers and the biopolymer matrix [56]. The limited effect of HMSNs may be associated with the chemical inertness and thermal stability of MSNs, which can retain their structure within polymer matrices without markedly disrupting the thermal behavior of the surrounding material [65]. Accordingly, HMSNs may act as thermally stable inorganic fillers without substantially affecting the thermal-transition behavior of the polymer matrix [66]. ULp-Caf@HMSNs showed no visible thermal transition below 45 °C (Figure 6c) under the HSM conditions used. This finding suggests that the nanocarrier structure was thermally stable over the tested temperature range; however, its stability within skin tissue requires further evaluation.

**Figure 7:**
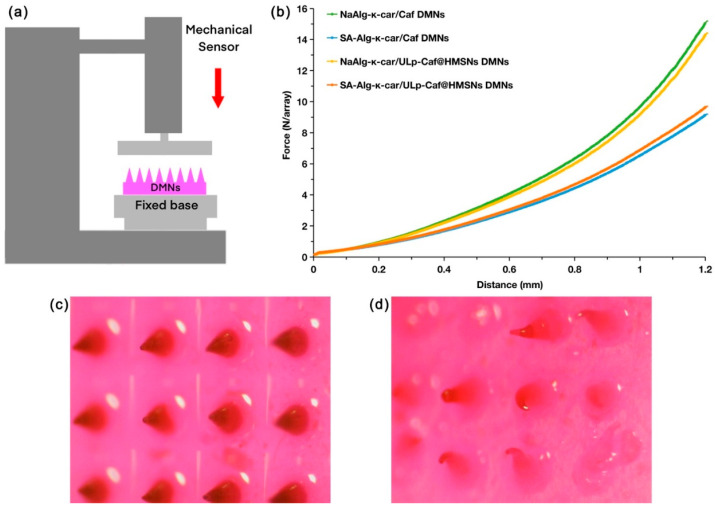
Mechanical characterization of the DMN formulations includes (**a**) a schematic of the universal tester used for compression testing, (**b**) force–displacement curves for each DMN formulation at a 0.5 mm displacement, (**c**) optical images before and (**d**) after compression (10 k-HA-MN) of RhB-loaded SA-Alg-κ-car/ULp-Caf@HMSNs DMNs.

Mechanical strength is an important parameter for DMNs because adequate resistance to compression is required for successful skin insertion. Successful transdermal drug delivery using DMNs requires a balance between sufficient mechanical strength and effective skin-insertion performance to overcome the resistance of the SC [67]. In this study, four DMN formulations were prepared and evaluated: NaAlg-κ-car/Caf, SA-Alg-κ-car/Caf, NaAlg-κ-car/ULp-Caf@HMSNs, and SA-Alg-κ-car/ULp-Caf@HMSNs. Mechanical testing was performed using a texture analyzer equipped with a calibrated load cell, as described in the experimental section. In the test setup (Figure 7a), each 100-needle DMN array was positioned with the needles oriented vertically. A parallel compression plate was then applied at a constant rate of 0.5 mm s^−1^. During compression, the force increased with displacement, and no distinct fracture point was observed. This response suggests that the arrays deformed progressively rather than failing abruptly under the applied load. Stereomicroscopic examination (Figure 7c,d) showed that SA-Alg-κ-car/ULp-Caf@HMSNs underwent vertical deformation and tip shortening after compression. These changes were consistent with plastic or viscoelastic deformation of the polymer matrix under mechanical loading. These results indicate that the NaAlg–κ-car-based formulations resisted higher compressive forces than the SA-containing formulations under the test conditions. Quantitative mechanical analysis (Figure 7b) showed that, within a displacement range of 0–1.2 mm, the NaAlg-κ-car/Caf and NaAlg-κ-car/ULp-Caf@HMSNs DMNs reached maximum compression forces of 15.1 and 14.3 N per 100-needle array, respectively. These values corresponded to 0.151 and 0.143 N per needle. In contrast, the SA-Alg-κ-car/Caf and SA-Alg-κ-car/ULp-Caf@HMSNs DMNs showed lower maximum compression forces, reaching 9.2 and 9.8 N per array, respectively. Taken together with the lower apparent thermal-transition temperatures, the reduced compression forces indicate that SA altered the organization of the polysaccharide matrix. However, the results do not suggest an overall improvement in matrix performance. Instead, SA produced a softer matrix with less resistance to compression under the tested conditions. This behavior may result from looser polymer-chain packing or changes in intermolecular interactions, although further structural analysis is needed to confirm the mechanism. The mechanical performance of the present formulations was comparable to that reported for other polymeric DMNs. Ando et al. found that PVA-based MNs exhibited fracture forces of approximately 0.107–0.192 N per needle, depending on the number of needles compressed during the test [68]. PVP/HPMC MNs showed compression forces of 0.14–0.19 N per needle before drug loading; after incorporation of an Oryza sativa L. extract complex, the values decreased to 0.08–0.12 N per needle [69]. In another study, acyclovir-loaded MNs prepared from Gantrez^®^ S-97 maintained their structural appearance after exposure to a compressive force of 0.089 N per needle for 30 s [70]. In the present study, the NaAlg–κ-car formulations exhibited maximum compression forces of 0.143–0.151 N per needle, whereas the SA-containing formulations showed lower values of 0.092–0.098 N per needle. These results are within the range reported for polymeric DMNs with adequate mechanical performance. Nevertheless, comparisons among studies should be interpreted cautiously because the measured force is affected by several experimental factors, including needle geometry, array density, the number of needles under compression, displacement, compression speed, and probe dimensions. The higher compression forces observed for the NaAlg-κ-car formulations may be related to closer polymer-chain packing and stronger intermolecular interactions within the alginate–carrageenan matrix, which could reduce free space between the polymer chains and improve structural integrity [71]. Notably, the incorporation of HMSNs affected the mechanical properties differently depending on the polymer matrix. NaAlg-κ-car/ULp-Caf@HMSNs DMNs showed slightly lower maximum compression forces than NaAlg-κ-car/Caf DMNs. In contrast, SA-Alg-κ-car/ULp-Caf@HMSNs DMNs showed slightly higher compression forces than the corresponding SA-Alg-κ-car/Caf formulation. These findings suggest that the effect of dispersed HMSNs on matrix integrity depends on the composition of the polymer system. The observed differences may be related to nanoparticle dispersion and interfacial interactions between the HMSNs and polymer chains [72].

### 2.2. Drug Release Behavior

The model with the best fit was identified based on the highest coefficient of determination (R^2^) (Table 4). The Korsmeyer–Peppas model showed the highest R^2^ values for both NaAlg-κ-car/Caf and SA-Alg-κ-car/Caf DMNs, indicating that it best described their Caf-release profiles. The diffusional exponent values (*n* = 0.45–0.89) were consistent with anomalous or non-Fickian transport, suggesting that Caf release was governed by a combination of diffusion through the hydrated polymer matrix and polymer swelling or relaxation [73]. This release mechanism may help maintain Caf availability over an extended period within the hydrated polymer matrix. In contrast, Caf@HMSNs, ULp-Caf@HMSNs, NaAlg-κ-car/ULp-Caf@HMSNs DMNs, and SA-Alg-κ-car/ULp-Caf@HMSNs DMNs were best described by the Higuchi model, which showed the highest R^2^ values among the models tested [74]. This finding suggests that Caf release was mainly governed by diffusion. According to the Higuchi model, drug release from a matrix is proportional to the square root of time, indicating that Caf was released primarily by diffusion through the porous carrier and polymer matrix. This model is commonly applied to drugs dispersed in insoluble or slowly eroding matrices and is therefore suitable for describing Caf release from these inorganic and hybrid systems [75].

**Figure 8:**
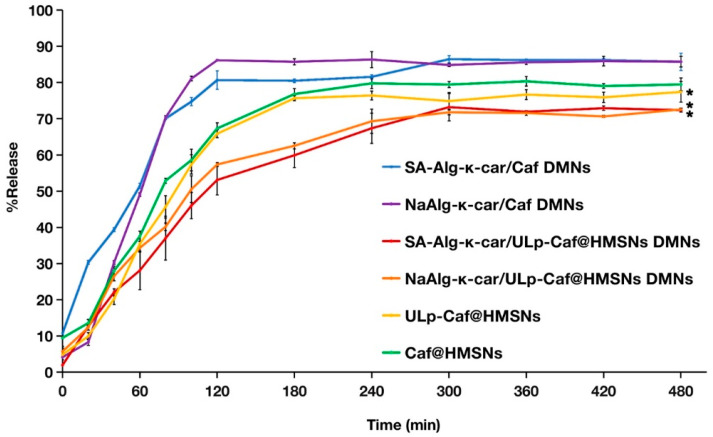
In vitro Caf release profiles of the samples in phosphate-buffered saline (pH 7.4) at 37.5 °C. Values are means ± standard deviation (*n* = 3). * *p* < 0.05 indicates significant difference from Caf@HMSNs DMNs.

PBS at pH 7.4 was used as a physiologically relevant aqueous release medium [76]. As demonstrated in Figure 8, after eight hours of release testing, the cumulative Caf release percentages were: NaAlg-κ-car/Caf DMNs (86%), SA-Alg-κ-car/Caf DMNs (86%), Caf@HMSNs (79%), ULp-Caf@HMSNs (77%), NaAlg-κ-car/ULp-Caf@HMSNs DMNs (73%), and SA-Alg-κ-car/ULp-Caf@HMSNs DMNs (72%). The effect of SA on Caf release depended on the formulation. For DMNs containing free Caf, the cumulative release after 8 h was similar with and without SA. In the ULp-Caf@HMSN formulations, SA caused only a slight reduction in Caf release compared with the corresponding unmodified formulation. These findings therefore do not support a consistent role of SA in improving or prolonging Caf release. Its effect appears to depend on the overall composition of the polymer matrix and the presence of the nanocarrier. All formulations showed an initial burst release during the first 3 h, possibly because of the rapid dissolution of Caf located near or on the formulation surface, together with the hydrophilic nature of the polymer matrix. However, formulations containing HMSNs showed a lower burst release than the polymer-only formulations [77]. NaAlg-κ-car/Caf DMNs and SA-Alg-κ-car/Caf DMNs exhibited faster initial release during the first 60 min, which may be associated with their higher swelling ratios. In the SA-modified formulations, the observed release behavior may be related to changes in polymer-chain organization and hydrophobic interactions introduced by SA. However, the present release data alone do not establish the exact mechanism.

Among the HMSN-containing formulations, Caf@HMSNs showed the highest percentage of Caf release compared with ULp-Caf@HMSNs, NaAlg-κ-car/ULp-Caf@HMSNs DMNs, and SA-Alg-κ-car/ULp-Caf@HMSNs DMNs. The lower release rates observed for the liposome-coated formulations may be attributed to the lipid coating, which forms an additional diffusion barrier around the HMSNs. This bilayer may partially block the mesoporous channels and act as a rate-limiting membrane, thereby slowing Caf diffusion from the silica matrix and producing a more sustained release profile [78]. Compared with the polymer-only formulations, the HMSN-containing systems showed slower Caf release under the present in vitro conditions. While NaAlg-κ-car and SA-Alg-κ-car matrices undergo rapid hydration and network relaxation, leading to faster drug diffusion, the silica framework is expected to hydrate and erode more slowly than the polymer matrix, which may contribute to the observed release profile [79]. The drug-release mechanism from HMSNs involves diffusion through the mesoporous network together with transport processes at the silica surface. This differs from the release behavior of hydrogel formulations, which is more strongly influenced by polymer swelling and dissolution. This difference may explain the faster Caf release from the polymer-based DMNs and the more prolonged release from the HMSN-containing systems. The liposomal coating may further regulate release by partially covering the pore openings and increasing diffusion resistance, thereby slowing the overall release rate [80]. The HMSN-containing formulations provided slower Caf release than the polymer-only systems under the tested conditions. Whether this release behavior improves intradermal or follicular drug exposure should be determined in future ex vivo permeation and deposition studies.

## 3. Materials and Methods

### 3.1. Chemicals and Materials

The chemicals and reagents used in this study included Caf, cetyltrimethylammonium bromide (CTAB), cholesterol, sodium carbonate, tetraethyl orthosilicate (TEOS), oleic acid, sodium alginate (100–200), *N*,*N*′-diisopropylcarbodiimide (C_7_H_14_N_2_), stearic acid, κ-carrageenan, and 4-dimethylaminopyridine (C_7_H_10_N_2_). These reagents were purchased from Tokyo Chemical Industry Co., Ltd. (Tokyo, Japan). Polysorbate^®^ 20 was supplied by Ajax Finechem Pty Ltd (Taren Point, Australia), whereas Phospholipon^®^ 90 G (phosphatidylcholine) was obtained from Lipoid GmbH (Ludwigshafen, Germany). Polydimethylsiloxane microneedle molds were purchased from MySkinRecipes^®^ (Bangkok, Thailand). Each mold measured 9 × 9 mm and contained a 10 × 10 array of conical cavities with a height of 600 μm, a base diameter of 300 μm, and a center-to-center spacing of 600 μm. All reagents were used as received without further purification.

### 3.2. Synthesis of SiO_2_ Nanoparticles

Silica nanoparticles were prepared by adding TEOS (10 mL) to a solution containing 95% ethanol (428 mL), water (60 mL), and NH_4_OH (10 mL) at 30 °C. The mixture was then stirred at 900 rpm for 2 h. The particles were subsequently collected by centrifugation at 15,000 rpm for 30 min using a centrifuge (Model 6000, KUBOTA, Osaka, Japan) and washed three times [12].

### 3.3. Fabrication of SiO_2_@CTAB-SiO_2_ Core/Shell Nanoparticles

A CTAB solution was prepared by dissolving 0.15 g of CTAB in a mixture of 30 mL of deionized water and 30 mL of ethanol. The solution was stirred at 900 rpm and 30 °C for 30 min. Subsequently, 0.55 mL of NH_4_OH and 0.1 g of SiO_2_ nanoparticles were dispersed in 20 mL of deionized water. After adding 0.25 mL of TEOS, the reaction was allowed to proceed for 6 h [45].

### 3.4. Targeted Etching Process for HMSN Fabrication

The SiO_2_@CTAB-SiO_2_ core–shell nanoparticles were collected by centrifugation at 15,000 rpm for 30 min, resuspended in 20 mL of deionized water, and stirred at 900 rpm for 10 h. Hollow mesoporous structures were then produced by adding Na_2_CO_3_ (0.47 g) and CTAB (2.04 g), followed by incubation at 50 °C for 10 h. To remove the template, the resulting particles were refluxed in a methanol/HCl mixture (50 mL/3 mL) at 80 °C for 24 h. The final HMSNs were collected by centrifugation at 15,000 rpm for 30 min and washed six times [12].

### 3.5. Caf Loading into HMSNs (Caf@HMSNs)

Caf-loaded HMSNs were prepared by dispersing HMSNs (0.1 g) in 50 mL of an aqueous Caf solution containing 0.1 g of Caf. The dispersion was stirred at 1000 rpm for 24 h at room temperature to promote Caf adsorption into the porous structure. The resulting particles were collected by centrifugation at 15,000 rpm for 10 min, washed twice with 2 mL of either ethanol or deionized water, and vacuum-dried at 60 °C for 2 h. The final product was designated Caf@HMSNs [45].

### 3.6. Preparation of Liposomal Dispersions (ULp-Caf@HMSNs)

#### 3.6.1. Preparation of Stock Solutions

Phospholipon^®^ 90 G (0.773 g) was dissolved in 5 mL of a chloroform/methanol mixture (2:1, *v*/*v*) under continuous stirring to prepare the lipid stock solution. Cholesterol (0.0618 g) was dissolved in 8 mL of the same solvent mixture. Polysorbate 20 (1.981 g) and oleic acid (0.991 g) were each dissolved separately in 2 mL of the chloroform/methanol mixture (2:1, *v*/*v*) using the same procedure.

#### 3.6.2. Preparation of ULps

To prepare ULp-Caf@HMSNs, 0.01 g of finely ground Caf@HMSNs was mixed with the prepared solutions of Phospholipon^®^ 90 G, cholesterol, Polysorbate 20, and oleic acid. The mixture was rotated under a stream of nitrogen to form a thin lipid film and then dried overnight in a desiccator. After 24 h, the film was rehydrated with phosphate buffer (pH 7.4) preheated to 80 ± 5 °C to a final volume of 10 mL. The resulting suspension was sonicated in an ice bath for 20 min at 40% amplitude using a 3 mm sonotrode (UP200S, Hielscher, Teltow, Germany). The particles were then collected by centrifugation at 15,000 rpm for 30 min and stored in a cool, dry place. The final product was designated ULp-Caf@HMSNs [45].

### 3.7. Encapsulation of Caf and ULp-Caf@HMSNs in NaAlg/κ-Car in the Complex Gel (NaAlg-κ-Car/Caf and NaAlg-κ-Car/ULp-Caf@HMSNs)

The incorporation of pure Caf and ULp-Caf@HMSNs into NaAlg/κ-car (NaAlg-κ-car) matrices was adapted from previously established protocols [81]. Pure Caf and ULp-Caf@HMSNs containing an equivalent amount of Caf were separately dispersed in 50 mL of deionized water and stirred at 500 rpm for 10 min. For the ULp-Caf@HMSNs formulation, the amount used corresponded to a net HMSN mass of 3 g. The dispersions were heated to 70 °C, and the ULp-Caf@HMSNs dispersion was additionally sonicated at 70 kHz for 20 min. κ-car (1 g) and NaAlg (1 g) were then added, and the mixtures were stirred until the polymers had completely dissolved. The temperature was subsequently reduced to 40 °C, and gelation was induced by the dropwise addition of 5 mL of 0.01 M NaCl while stirring at 60 rpm. The gels were maintained under these conditions at 40 °C for 1 h to allow further gel formation and stabilization. The final formulations were designated NaAlg-κ-car/Caf and NaAlg-κ-car/ULp-Caf@HMSNs, respectively.

### 3.8. Preparation of Caf and ULp-Caf@HMSNs in Alginate Stearate/κ-Car in the Complex Gels (SA-Alg-κ-Car/Caf and SA-Alg-κ-Car/ULp-Caf@HMSNs)

Using a mortar and pestle, SA (0.56 g) and N,N′-diisopropylcarbodiimide (DIC; 0.205 mL) were combined and ground for 3 min. The NaAlg-κ-car/Caf or NaAlg-κ-car/ULp-Caf@HMSNs formulation was then added, followed by 4-dimethylaminopyridine (DMAP; 0.254 g). For the ULp-Caf@HMSNs formulation, the amount used corresponded to a net HMSN mass of 3 g and contained an equivalent amount of Caf. The reaction mixture was ground for an additional 30 min. The resulting material was washed three times with ethanol (2 mL per wash), producing a white paste. The paste was then centrifuged at 15,000 rpm for 30 min. The precipitate was collected and dried at 28 °C for 48 h. The final products were designated SA-Alg-κ-car/Caf and SA-Alg-κ-car/ULp-Caf@HMSNs, respectively [82].

### 3.9. Preparation of NaAlg-κ-Car/Caf DMNs, SA-Alg-κ-Car/Caf DMNs, NaAlg-κ-Car/ULp-Caf@HMSNs DMNs and SA-Alg-κ-Car/ULp-Caf@HMSNs DMNs) in the Complex Thermosensitive DMNs

**Figure 9:**
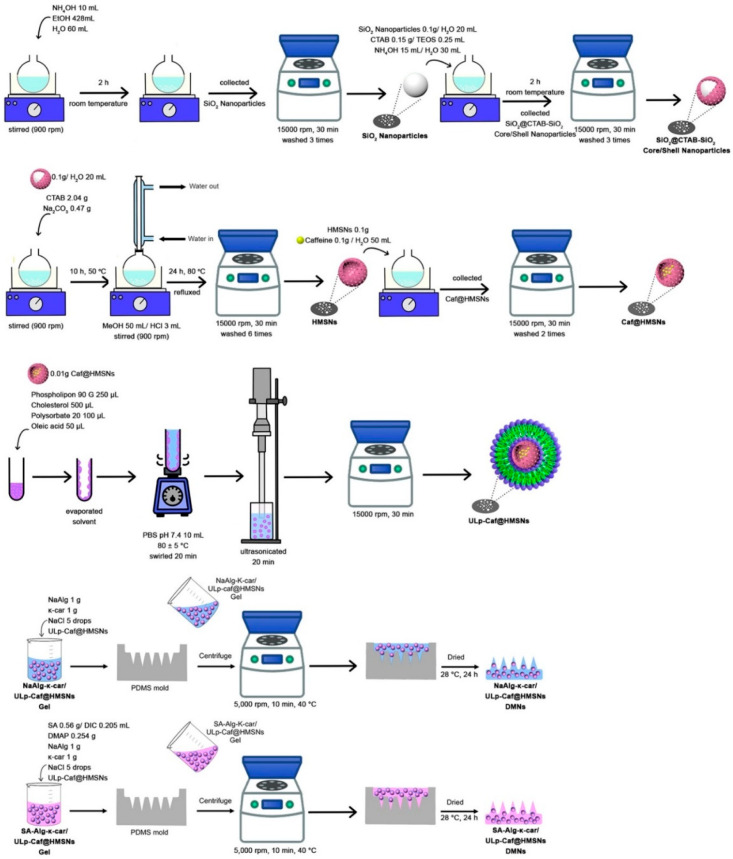
Schematic diagram of the preparation of HMSNs, Caf@HMSNs, ULp-Caf@HMSNs, NaAlg-κ-car/ULp-Caf@HMSNs DMNs, and SA-Alg-κ-car/ULp-Caf@HMSNs DMNs. (Except for the case of NaAlg-κ-car/Caf DMNs and SA-Alg-κ-car/Caf DMNs, Caf is used in place of ULp-Caf@HMSNs.).

The procedure for preparing thermosensitive DMNs containing NaAlg-κ-car/Caf, SA-Alg-κ-car/Caf, NaAlg-κ-car/ULp-Caf@HMSNs, or SA-Alg-κ-car/ULp-Caf@HMSNs was adapted from the method described by Kim et al. [83]. One milliliter of the corresponding gel formulation was applied to the surface of each polydimethylsiloxane (PDMS) mold. Each formulation contained either 0.06 g of HMSNs or an equivalent amount of Caf. The filled molds were then heated in an oven at 40 °C, allowing the gel to become sufficiently fluid for molding. To ensure complete filling of the mold cavities, the formulations were centrifuged at 5000 rpm for 10 min at 40 °C. After centrifugation, the formulations were dried at 28 °C for 24 h to form solid microneedle arrays. The resulting DMNs were then carefully removed from the PDMS molds. The formulations were designated NaAlg-κ-car/Caf DMNs, SA-Alg-κ-car/Caf DMNs, NaAlg-κ-car/ULp-Caf@HMSNs DMNs, and SA-Alg-κ-car/ULp-Caf@HMSNs DMNs, according to their respective compositions. The preparation of HMSNs, Caf@HMSNs, ULp-Caf@HMSNs, NaAlg-κ-car/ULp-Caf@HMSNs DMNs, and SA-Alg-κ-car/ULp-Caf@HMSNs DMNs is schematically illustrated in Figure 9. The coating of Caf@HMSNs with ULps and the preparation of the NaAlg-κ-car and SA-Alg-κ-car composite gels followed previously reported methods, with Caf loading into HMSNs performed as described in the present study. For the NaAlg-κ-car/Caf DMNs and SA-Alg-κ-car/Caf DMN formulations, Caf was incorporated directly as the active compound instead of ULp-Caf@HMSNs.

### 3.10. Characterization of Caf@HMSNs, ULp-Caf@HMSNs, NaAlg-κ-Car/Caf, NaAlg-κ-Car/ULp-Caf@HMSNs, SA-Alg-κ-Car/Caf and SA-Alg-κ-Car/ULp-Caf@HMSNs in the Complex Gels and DMNs

The morphology of the samples was characterized by TEM (JEOL, Tokyo, Japan) and FESEM (JSM-7800F, JEOL, Tokyo, Japan). The FTIR spectra were recorded using an FTIR spectrometer (Nicolet iS50, Thermo Fisher Scientific, Madison, WI, USA) and the KBr pellet method. Spectra were collected over the range of 4000–400 cm^−1^ at a resolution of 4 cm^−1^, with 64 scans averaged for each measurement. The hydrodynamic particle size, polydispersity index (PDI), and zeta potential of HMSNs and ULp-Caf@HMSNs were measured using a Zetasizer Nano ZS (Malvern Panalytical Ltd., Malvern, UK). Before measurement, each sample was dispersed in deionized water and diluted to an appropriate concentration to minimize multiple scattering. The dispersions were gently mixed and briefly sonicated to reduce particle aggregation. Particle size and PDI were determined by dynamic light scattering. Zeta potential was calculated from the measured electrophoretic mobility using the model selected in the instrument software. Measurements were performed at 25 °C after equilibration in the instrument. Zeta-potential values were calculated using the Smoluchowski approximation. N_2_ adsorption–desorption measurements were performed at −196 °C using a 3Flex analyzer (3Flex analyzer, Micromeritics Instrument Corporation, Norcross, GA, USA) to determine the surface area and porosity of the HMSNs. The specific surface area was calculated using the BET method, whereas the pore-size distribution was determined using the BJH method.

### 3.11. External Appearance and Morphology

Morphological examination of the specimens was performed using hot-stage microscopy (HSM) with an optical microscope fitted with a METTLER TOLEDO hot stage (HS1 and HS82, METTLER TOLEDO, Greifensee, Switzerland). All DMN formulations were placed on glass slides and heated from 30 to 45 °C at a controlled rate of 0.5 °C min^−1^. Structural changes during heating were recorded using OLYMPUS Stream Basic software (version 2.5; Olympus Soft Imaging Solutions GmbH, Münster, Germany). The dimensional parameters of all DMN formulations, including needle-base diameter and interneedle spacing, were measured using a Leica microscope (Leica Microsystems, Milton Keynes, UK).

### 3.12. Determination of Mechanical Characteristics

The mechanical properties of the DMN formulations were evaluated using a texture analyzer (TA-XTplusC, Stable Micro Systems Ltd., Godalming, UK) equipped with a 5 kg load cell. Thumb pressure was simulated by applying a compression force of 32 N using a cylindrical probe (P/0.5). The trigger force was set at 0.049 N, while the pre-test, test, and post-test speeds were 1, 0.5, and 10 mm s^−1^, respectively. Force–displacement profiles were recorded, and microneedle heights before (Hb) and after (Ha) compression were measured using a digital microscope. The percentage reduction in height was then calculated using the following Equation (1) [84,85]:

% Height reduction = (Hb − Ha) / Hb × 100%(1)

### 3.13. Drug Release Behavior

In vitro Caf release was evaluated using 1 mL of each DMN formulation or 0.06 g of each HMSN-based formulation, with all samples containing equivalent amounts of Caf. The samples were placed in 30 mL of PBS (pH 7.4) and agitated at 100 rpm. The tested formulations included Caf@HMSNs, ULp-Caf@HMSNs, NaAlg-κ-car/Caf DMNs, SA-Alg-κ-car/Caf DMNs, NaAlg-κ-car/ULp-Caf@HMSNs DMNs, and SA-Alg-κ-car/ULp-Caf@HMSNs DMNs. At predetermined time points between 0 and 480 min, 100 μL samples were withdrawn and replaced with an equal volume of fresh PBS. The Caf concentration was determined by HPLC after appropriate dilution and filtration through a 0.2 μm membrane filter.

The measured Caf concentration was used to calculate the amount released at each time point (Mc). The corrected cumulative amount released (Mc′) was calculated by accounting for the Caf removed during previous sampling, as described by Hodali et al. [86]. The cumulative percentage release was then calculated using Equation (2).

% Release = (Mc′/Cf) × 100(2)

where Cf is the Caf content of each formulation.

Experimental data were analyzed using kinetic models to determine release behavior. In Equation (3), the zero-order kinetic model is expressed [10,87].

Mc′ = k0t(3)

In Equation (4), the first-order kinetic model is expressed [88].

ln(Cf-Mc′) = lnCf − k1t(4)

In Equation (5), the Higuchi model is expressed [10,87].

Mc′ = kHt0.5(5)

In Equation (6), the Korsmeyer–Peppas model is expressed [45,89].

Mc′/Cf = ktn(6)

In these models, Cf represents the total Caf content loaded into the HMSNs, whereas Mc′ is the cumulative amount of Caf released at time t. The release-rate constant is denoted by k_0_, k_1_, k_H_, or k, depending on the kinetic model applied, while n represents the diffusional exponent associated with the drug-release mechanism. The Caf-release profiles of the HMSN-based formulations were fitted to these kinetic models. In general, the release rate may depend on the amount of Caf remaining within the mesoporous structure [86].

### 3.14. High-Performance Liquid Chromatography (HPLC) Analysis

Caf content was determined using an HPLC system (UFLC, Shimadzu Corporation, Kyoto, Japan) equipped with a C18 column and a UV detector set at 280 nm. The mobile phase consisted of 0.5% acetic acid in water and methanol (50:50, *v*/*v*) and was delivered isocratically at a flow rate of 0.8 mL min^−1^. A 10 μL aliquot of each sample was injected, and the analysis was performed at room temperature.

#### Entrapment Efficiency (%EE) and Loading Capacity (%LC)

The loading capacity (%LC) and encapsulation efficiency (%EE) of Caf@HMSNs were determined as follows. The solid particles were separated from the supernatant by centrifugation at 15,000 rpm for 30 min. After one wash with deionized water, the solid fraction was transferred to a desiccator and dried overnight at 60 °C. Before analysis, the supernatant was filtered through a 0.22 μm Millipore membrane filter. The Caf concentration in the supernatant was then quantified by HPLC.

The %EE and %LC were calculated using Equations (7) and (8), as described in Subongkot et al. [90].

%EE = (Cf/Ci) × 100(7)

The initial amount of Caf introduced into the HMSNs is represented by Ci, while the amount of Caf encapsulated within the HMSNs is denoted by Cf.

%LC = (Cf/H) × 100(8)

The amount of Caf@HMSNs is indicated by H, and Cf represents the quantity of Caf entrapped within the HMSNs.

### 3.15. Statistical Analysis

The mean ± SD of each of the three experiments (*n* = 3) is displayed. Statistical significance was determined using one-way ANOVA and Tukey’s post hoc multiple comparison test, with *p*-values < 0.05.

## 4. Conclusions

This study successfully developed thermosensitive DMNs incorporating ULp-Caf@HMSNs as a controlled-release platform for Caf. The synthesized HMSNs exhibited a high BET-specific surface area of 1015.44 m^2^ g^−1^, a pore volume of 1.02 mL g^−1^, and an average pore diameter of approximately 1.8 nm. This pore diameter lies near the boundary between the microporous and mesoporous ranges. The relatively narrow pores may help regulate Caf diffusion, while the hollow internal structure provides additional space for drug loading. FTIR analysis supported the successful formation of the developed systems. Caf retained its characteristic bands at 1691 and 1641 cm^−1^ after loading, while alginate and κ-car showed their typical polysaccharide bands. The DMN formulations exhibited thermal transitions at approximately 33–35 °C, demonstrating temperature-dependent structural behavior within the tested range. Mechanical characterization revealed superior performance of NaAlg-κ-car systems, which showed compression forces of 14.3–15.1 N per array, comparable to values reported for mechanically competent DMNs. Drug-release analysis revealed distinct kinetic profiles. The conventional hydrogel formulations exhibited non-Fickian transport and were best described by the Korsmeyer–Peppas model (R^2^ > 0.96), whereas the HMSN-containing systems followed Higuchi diffusion-controlled release (R^2^ > 0.97). The HMSN-based formulations showed a more sustained release profile, with approximately 72–73% of Caf released over 6 h, compared with the faster release from the conventional hydrogel systems. This behavior suggests that the HMSN-containing systems may prolong Caf release while reducing the magnitude of the initial burst. The combination of HMSNs and a liposomal coating offers two complementary functions: the hollow mesoporous structure provides substantial space for Caf loading, while the lipid coating further regulates its release. The temperature-responsive behavior may be useful for future development of a user-applied delivery platform. Overall, the developed platform showed suitable physicochemical, mechanical, thermal, and in vitro release properties, providing a basis for further evaluation as a minimally invasive topical or transdermal Caf-delivery system. However, ex vivo skin insertion, permeation, and deposition studies are required before conclusions can be drawn regarding transdermal or follicular delivery.

## 5. Patents

Portions of this content have been submitted for a petty patent application in Thailand under application number 2503001846.

## Figures and Tables

**Table 1:** Principal FTIR vibration bands of the raw materials, nanoparticles, and composite formulations.

Sample	Principal Band(s) (cm^−1^)	Main Assignment
HMSNs	3200–3450	O–H stretching of surface silanol groups and adsorbed water
HMSNs	1061	Asymmetric Si–O–Si stretching
Caf	1691 and 1641	C=O stretching and coupled xanthine-ring vibrations
Caf	744	Out-of-plane deformation of the xanthine ring
Caf@HMSNs	1675	Caf carbonyl-associated vibration
Caf@HMSNs	1038	Shifted asymmetric Si–O–Si stretching
κ-car	1210	S=O stretching of sulfate ester groups
κ-car	922 and 833	3,6-Anhydrogalactose and C–O–S vibrations
NaAlg	1590 and 1394	Asymmetric and symmetric COO^−^ stretching
NaAlg	1010	C–O and C–O–C stretching of the polysaccharide backbone
SA	2913 and 2846	Asymmetric and symmetric CH_2_ stretching
SA	1698	C=O stretching of the carboxylic acid group
NaAlg–κ-car/Caf and SA–Alg–κ-car/Caf	1686–1636	Overlapping Caf carbonyl/ring and polymer-associated vibrations
Final ULp-Caf@HMSN-loaded composites	Retained silica-, lipid-, Caf-, and polysaccharide-associated bands	Overlapping vibrations of the individual components

**Table 2:** Particle size, zeta potential, and PDI of the samples.

Formulation	Size (nm)	PDI	Zeta Potential (mV)
HMSNs	198.2 ± 17.66	0.487 ± 0.07	−17.9 ± 2.26
ULp-Caf@HMSNs	243.9 ± 20.67	0.422 ± 0.11	−24.6 ± 3.44

Values are the mean ± standard deviation (*n* = 3).

**Table 3:** The average diameter, base distance, and melting point of the DMN formulations.

Formulation	Melting Point (°C)	Average DMN Diameter (μm)	Average DMN Base Distance (μm)
NaAlg-κ-car/Caf DMNs	35.09 ± 0.5	295.71 ± 0.89	487.45 ± 5.43
SA-Alg-κ-car/Caf DMNs	34.09 ± 0.5	286.83 * ± 3.41	498.21 * ± 2.18
NaAlg-κ-car/ULp-Caf@HMSNs DMNs	35.09 ± 0.5	291.96 ± 2.98	491.17 ± 1.86
SA-Alg-κ-car/ULp-Caf@HMSNs DMNs	33.09 ± 0.5	317.22 ± 2.59	501.46 ± 5.50

Data are expressed as mean ± SD (*n* = 3). * is the average DMN diameter and base distance that *p* < 0.05 when compared to NaAlg-κ-car/Caf DMNs.

**Table 4:** Caf release and kinetic parameters.

Formulation	% Release at 8 h	Ct (mg)	Zero-Order k	Zero- Order R^2^	First-Order k	First- Order R^2^	Higuchi k	Higuchi R^2^	Korsmeyer– Peppas k	Korsmeyer– Peppas R^2^	*n*
NaAlg-κ-car/Caf DMNs	86 ± 1.50	19.35 ±0.34	0.16	0.6218	0.0079	0.4901	3.42	0.8047	0.038	0.973	0.6207
SA-Alg-κ-car/Caf DMNs	86 ± 2.38	19.37 ± 0.54	0.17	0.7165	0.0049	0.5184	2.81	0.8786	0.12	0.9647	0.5918
Caf@HMSNs	79 ± 1.76	17.80 ± 0.4	0.25	0.8069	0.0063	0.6483	4.93	0.9766	0.088	0.8772	0.4315
ULp-Caf@HMSNs	77 ± 2.84	17.40 ± 0.64	0.25	0.7857	0.0077	0.6115	5.05	0.9886	0.047	0.9072	0.5526
NaAlg-κ-car/ULp-Caf@HMSNs DMNs	73 ± 2.32	16.50 ± 0.52	0.13	0.848	0.0067	0.6255	2.6	0.973	0.065	0.9451	0.4859
SA-Alg-κ-car/ULp-Caf@HMSNs DMNs	72 ± 2.52	16.20 ± 0.57	0.14	0.8954	0.0084	0.56	2.7	0.9929	0.027	0.973	0.6594

Each value represents the mean ± SD (*n* = 3). The release profiles of ULp-Caf@HMSNs, NaAlg-κ-car/ULp-Caf@HMSNs DMNs, and SA-Alg-κ-car/ULp-Caf@HMSNs DMNs were significantly different from that of Caf@HMSNs (*p* < 0.05).

## Data Availability

Data is contained within the article.

## Abbreviations

The following abbreviations are used in this manuscript:

Caf	Caffeine
HMSNs	Hollow mesoporous silica nanoparticles
MNs	Microneedles
MSNs	Mesoporous silica nanoparticles
DMNs	Dissolving microneedles
ULp	Liposomes
NaAlg	Sodium alginate
SA	Steric acid
SA-Alg	Alginate stearate
κ-car	κ-Carrageenan
NaAlg-κ-car	A system comprising Sodium alginate and κ-car
SA-Alg-κ-car	A system comprising Alginate stearate and κ-car
ULp-Caf@HMSNs	Caf-loaded HMSNs coated with ULps
NaAlg-κ-car/Caf	A system comprising Sodium alginate and κ-car encapsulating pure Caf
NaAlg-κ-car/ULp-Caf@HMSNs	A system comprising Sodium alginate and κ-car encapsulating ULp-Caf@HMSNs
SA-Alg-κ-car/Caf	A system comprising Alginate stearate and κ-car encapsulating pure Caf
SA-Alg-κ-car/ULp-Caf@HMSNs	A system comprising Alginate stearate and κ-car encapsulating ULp-Caf@HMSNs
NaAlg-κ-car/Caf DMNs	A system comprising sodium alginate and κ-car prepared as DMNs for Caf loading
NaAlg-κ-car/ULp-Caf@HMSNs DMNs	A system comprising Sodium alginate and κ-car prepared as DMNs for ULp-Caf@HMSNs loading
SA-Alg-κ-car/Caf DMNs	A system comprising Alginate stearate and κ-car prepared as DMNs for Caf loading
SA-Alg-κ-car/ ULp-Caf@HMSNs DMNs	A system comprising Alginate stearate and κ-car prepared as DMNs for ULp-Caf@HMSNs loading
CTAB	Cetyltrimethylammonium bromide
PBS	Phosphate-buffered saline
TEOS	Tetraethoxysilane
EtOH	Ethanol
MeOH	Methanol
DLS	Dynamic light scattering
%EE	% Entrapment efficiency
%LC	% Loading capacity
